# A Scenario Analysis for Implementing Immunocastration as a Single Solution for Piglet Castration

**DOI:** 10.3390/ani12131625

**Published:** 2022-06-24

**Authors:** Li Lin-Schilstra, Paul T. M. Ingenbleek

**Affiliations:** 1 College of Economics and Management, Nanjing Agricultural University, Nanjing 210095, China; 2 Marketing and Consumer Behaviour Group, Wageningen University & Research, 6706 KN Wageningen, The Netherlands; paul.ingenbleek@wur.nl

**Keywords:** animal welfare, European market, boar taint, gonadotrophin-releasing factor, pig meat

## Abstract

**Simple Summary:**

The 2010 European Declaration aimed at abandoning surgical castration and switching to alternative methods. With an intention to facilitate the internal trade, the European Union (EU) was initially in favor of a single alternative. Immunocastration, as an active vaccine against the Gonadotropin Releasing Hormone, was proposed as a potential solution. However, its market potential is not yet well understood. This study applied the method of scenario analysis to examine whether and under what conditions immunocastration could be the general solution sought by the EU. Specifically, it developed two extreme scenarios. Both scenarios suggest that it is unlikely that immunocastration will become a single solution for all producers in the EU.

**Abstract:**

Painful castration of male piglets to avoid boar taint can potentially be replaced by three more ethical alternatives: entire male production in combination with a detection method, immunocastration (an active vaccination against the gonadotrophin-releasing factor, GnRF), and castration with pain relief (anesthesia and/or analgesia). With the aim of abandoning piglet castration and facilitating internal trade, the European Union (EU) was initially in favor of a single alternative. Immunocastration was proposed as a potential solution, but it has not yet been sufficiently assessed regarding its market potential. To address this point, this paper uses scenario analysis to examine whether and under what conditions immunocastration could be the general solution sought by the EU. The study constructs two extreme scenarios: one in which all uncertain elements negatively influence the growth of immunocastration; another in which all uncertain elements have positive influences. These scenarios provide insights into the variance in possible futures for the implementation of immunocastration. The results show that it is unlikely that immunocastration will become a single solution for all producers in the EU, because it is not the optimal solution for all types of EU pork production systems (i.e., cost-efficiency oriented, quality oriented, animal-friendly oriented, import dependent). Rather than debating and looking for evidence about which single method is the best for the entire EU, EU authorities are advised to allow the co-existence of all alternatives and to develop protocols for applying them in the pork industry.

## 1. Introduction

In many countries, ethical considerations concerning the origin of animal products have arisen over the past decades [1]. In the European Union (EU), the European Commission performs surveys on EU citizens’ opinions and expectations about farm-animal welfare [2], based on which it drafts legislation to address animal-welfare issues and to improve the conditions of farm animals, such as dairy cows, laying hens, and pigs [3]. Piglet castration is one of the issues that has attracted public attention [4].

Piglet castration is performed to avoid undesired behaviors such as aggression and to prevent the occurrence of an unpleasant odor in meat, known as boar taint, a smell that many consumers find repulsive [5]. Studies have shown that piglet castration, even performed soon after birth, can cause tremendous pain, incurring strong public disapproval [6]. Although farmers can apply anesthesia and analgesia during and after the procedure, these are not routinely applied [7]. An alternative method is to raise entire males with specific feeding compounds and detection methods in the slaughter line. This option is limited in some European countries because some producers are concerned about the effectiveness of the detection method and the quality of meat from entire male pigs. Another option is immunocastration, which is undertaken by injecting vaccines against GnRH (immunocastration is active immunization against the gonadotropin-releasing hormone (GnRH), a key hormone of the endocrine cascade regulating reproductive functions; the treatment requires at least two injections of Improvac^®^ vaccines during the fattening period) pulses to disrupt testis growth and steroid synthesis, thereby lowering the risk of boar taint occurring. This method is widely used in countries such as Australia, New Zealand, and Brazil, and its use has been approved in Europe since 2009 [8].

However, the practical usage of immunocastration is still limited in Europe. The successful implementation of immunocastration does not rely solely on farmers. Farmers implementing immunocastration have concerns about safety risks during vaccination; thus, training is needed for farmers [9]; processors and slaughterhouses have to agree to accept immunocastrated carcasses at a reasonable price [10]; for processors and slaughterhouses, it is important that retailers and consumers accept immunocastrated pork products. Furthermore, factors such as consumers’ knowledge about this castration issue [11], attitudes toward vaccines [12], and willingness to pay extra [13] are believed to be crucial in marketing immunocastrated products. These factors, which involve multiple supply chains and other stakeholders (e.g., special interest groups and governments), do not combine in any simple and straightforward way that can be easily predicted and prepared, as interactions between all these factors lead to a large number of potential future directions. Each of these futures requires a different type of support for the implementation of immunocastration.

The aim of this article is to explore potential futures by applying the scenario analysis method. Scenario analysis is a systemic technique to create a better understanding of potential futures [14]. For this, the method guides researchers to identify factors that may be crucial in influencing the future. Rather than predicting the future, it helps policymakers and business planners to learn how the future could be, accepting its complexities and uncertainties. To that end, two scenarios are developed: one in which the uncertainties turn out to be favorable, and one in which they are all unfavorable, for immunocastration as a potential single solution. If policymakers and business planners have an understanding of potential futures, they are better equipped to deal with problems that have a broad scope [14].

## 2. Materials and Methods

In different domains, decision makers constantly face challenges of knowing the future. Traditional planning based on historical trends is not appropriate to explore the options for addressing the challenges posed by highly complex and uncertain problems. To address the challenge, decision makers are advised to use the method of scenario analysis. The approach embraces future ambiguity with analytical rigor of available models [15]. It has been evidenced to be effective in dealing with uncertainties. Researchers using scenario analysis systematically generate a number of contrasting futures that diverge from the historical trend [16]. The scenario analysis method, initially developed by Kahn and Wiebe to determine possible outcomes of nuclear war between the USSR and the USA [17], became famous when it led Shell to adjust its business management to hedge against the oil crisis in the 1970s. It has now become a standard approach to assess wicked problems, such as animal-welfare assessment [18], global food safety [19], and environmental challenges [20].

The purpose of scenario analysis is to improve current decisions on the basis of a better appreciation of the range of potential futures, referred to as scenarios. Scenarios are “a description of potential future conditions, developed to inform decision-making under uncertainty” [21]. It should be noted that trends, expert predictions, and quantitative modeling are all parts of scenario-building exercises but not scenarios themselves. To be noticed, the approach does not aim to predict the future. Instead, the development of scenarios copes with uncertainty by presenting a range of plausible futures, without necessarily assigning probabilities to the outcomes. Scenarios encompass a realistic projection of current trends and predictions. Their value lies in capturing key aspects of uncertainty about the future of a system and in stimulating people to evaluate and reassess their beliefs about the system [22].

There are many different approaches to scenario planning, with various formats, frameworks, and methodological descriptions. The scenario analysis steps suggested by Schoemaker are a generic, synthesized method for normative scenario building [14]. Normative scenarios are intended to meet a specific target (in our case, the implementation of immunocastration as a single solution). The focus of normative scenario building is on exploring and understanding the interrelationships among trends and key uncertainties that are not controllable by the decision makers. Because we aim to understand the potential variance of the futures, we develop so-called ‘forced’ or ‘extreme’ scenarios. Such scenarios are helpful in reducing dilemmas of legitimacy in futures’ analysis [23]. They are also particularly helpful to understand the range of potential futures and thus help to identify strategic issues [18].

To ensure the validity of scenario analysis, we built on prior information from the literature, including publications and research reports, regarding the piglet castration issue and its alternatives. Additionally, we organized discussions during consortium meetings in the context of the SuSI project in December 2019 and January 2021, respectively. Seventeen experts from seven countries participated in the consortium meetings. They have diverse backgrounds in academia and industry, including the area of nutrition management, animal welfare evaluation, on-farm management and product quality assessment, and consumer acceptance, etc. For participants to start with, the mediators (authors) first presented insights drawn from early desk research. All participants were then invited to comment on and validate the prepared inputs. They were free to elaborate or add extra inputs and to discuss with each other. We made notes of the discussion and drafted preliminary scenarios. The discussion was ended with summaries of opinions during the meeting. By doing so, we ensured internal consistency and plausibility of our scenarios. Following Schoemaker’s steps (please refer to Appendix A), we developed two representative and extreme positive and negative scenarios. Although neither scenario is likely to materialize, they help to understand the range of potential futures and thus call for practitioners to reassess their strategies.

### 2.1. Step 1 Define the Issue

The first step in scenario analysis is to define the issue at hand in terms of timeframe, scope, and decision variables, based on reviews of uncertainty and volatility. The issue here was the implementation of immunocastration as a single alternative in the EU market.

In 2010, the European declaration on alternatives to surgical castration of pigs was signed voluntarily by 33 stakeholders from the whole pork chain (including scientists, veterinarians, and animal welfare NGOs). The declaration stipulates that from 1 January 2012, surgical castration of pigs shall be performed only with prolonged analgesia and/or anesthesia, and from 2018 surgical castration of pigs should be phased out altogether. The deadline to phase out castration has passed. The 2012 goal, surgical castration with analgesia and/or anesthesia, was not achieved, not to mention the complete cessation of surgical castration. The choice of alternatives includes: castration with analgesia and/or anesthesia, entire males, and immunocastration. During the past decade, several projects were undertaken, including CASTRUM, PIGCAS, Boarcheck, Campig, and ALCASDE. Researchers evaluated the pros and cons of alternatives, ascertained stakeholders’ preferences, provided guidance for farm management, and assessed consumers’ acceptance of the alternative methods of castration with pain relief, entire males, and immunocastration. The findings provided policymakers with evidence on how to regulate the market and help practitioners to determine the best alternative for their business. Arriving at a best alternative as a single harmonized solution would benefit the EU single market because it would mean fewer barriers to internal trade [4].

The most recent EU project on immunocastration was SuSI (sustainability in pork production with immunocastration), which started in September 2017. The SuSI project’s last meeting was in January 2020, and the project was projected to be delivered by September 2021. Therefore, the period that we explore in developing the scenarios is the subsequent time period between 2021 and 2026. The implementation of immunocastration is seen as a process in which stakeholders have to favor, agree upon, or collaborate for immunocastration. For the SuSI project’s purposes, the scenarios’ scope is restricted to the EU.

### 2.2. Step 2 Identification of Actors

The second step in scenario analysis is usually to identify actors. According to the stakeholder literature, firms directly involved in the value-producing process, for instance farmers, processors, and slaughterhouses in the pork production chain, are easily defined as primary stakeholders. Scholars have, however, focused also on groups not directly involved in production, such as animal-interest groups, communities, and media. These are often referred to as secondary stakeholders [24].

Trienekens et al. [25] identified the major stakeholders in most European pork chains. In our case, the major stakeholders are farmers, because they are the ones who actually implement immunocastration when raising pigs; slaughterhouses/processors, because they may still need to implement a detection method to sort out carcasses in case vaccination has been ineffective (the reasons for poor immune response are not clear, Kress et al., 2020); retailers, because they can make and influence important decisions regarding the criteria for the sourcing of meat products. As a group, they make purchase decisions about products that vary in how they are produced. Governments (including the European Commission and member state governments) play an important role in shaping the policies, rules, and legislation that set the conditions for the implementation of animal-friendly methods for chain members and consumers. Chain members and consumers are also influenced by special interest groups, including animal-interest groups and media that publicize their ideals to the industry and governments. Veterinarians may be approached by the government to advise on vaccine usage, and they may be appointed to administer drugs. In some countries, administration of anesthesia or vaccines forms part of veterinarians’ job descriptions. Scientists endeavor to find practical methods to help the industry, and pharmaceutical companies provide the vaccines to users.

Studies have shown that the conflicts regarding which alternative to choose go beyond stakeholder groups or geographical borders [4]. Furthermore, even members of the same stakeholder group may not share the same opinion, whereas members in different countries may agree with one another. Lin-Schilstra and Ingenbleek’s findings based on comparative case studies suggested that the main reason for conflicts is related to differences in production systems. Stakeholders are generally willing to switch from painful castration to one of the alternatives, but their specific preferred alternatives are strongly dependent on the structure, scale, and cost and quality orientation of their production system [4]. For instance, farmers in integrated production chains that are oriented toward cost efficiency generally support entire male production (e.g., farmers in the Netherlands, in northern Germany, or integrated into Cooperl, France), whereas farmers in production chains oriented toward high quality show more interest in castration with pain relief or immunocastration [26].

Following this line of reasoning, we classify actors according to the orientation of their production systems. From previous studies [4], we identified five production systems. Specifically, when a system is oriented toward cost efficiency, which often includes export-oriented systems such as the major production in the Netherlands, Spain, and Cooperl in France, producers tend to favor the entire male solution (Denmark is one of the largest pork exporters in the EU. However, less than 2% of pigs are raised uncastrated. The Danish pork industry managed to transit to entire male production during the early 1990s. A trading conflict between Denmark and Germany led Danish producers to revert to the castration method. Ever since, most male piglets in Denmark are castrated under analgesia (around 95%)); when the system is oriented toward high quality, the meat taste is often emphasized, for example, chains in France and Italy [27], producers tend to favor castration with pain relief; when the system is oriented toward regional specialties, producers in some small local chains in France might be interested in immunocastration [4]; when the system focuses strongly on improving animal welfare, such as in Sweden or Norway, producers tend to support castration with both anesthesia and analgesia [7]; when the system is peripheral in the European market, which often relies on imported meat from counties such as Slovenia [28], producers are less active in debating the castration issue, not to mention choosing a certain method. It should be noted that in some countries several types of production systems co-exist. For instance, in northern Germany, the dominant production system is oriented toward cost efficiency, whereas, in southern Germany, the production system is oriented toward special regional products and high quality [29]. Members of the same stakeholder group might hold different positions because of their attachment to a different production system. Table 1 provides an overview of segmentation in which stakeholder groups are identified across different production systems. As the major conflicts about accepting immunocastration lie in differences in production systems, our scenario development focuses on systems as actors.

### 2.3. Step 3 Trends: Pre-Determined Elements That Significantly Affect the Variables of Interest

In the third step, potential trends or pre-determined elements that are considered to have the potential to affect the implementation of the issue at hand are identified. In the pig castration case, these include:

A growing societal awareness of animal welfare (although the awareness level differs among member states, later referred to as MSs) led by special interest groups [30];
An increased market acceptance of meat replacers because of the growing varieties of meat analogues and in vitro meat. Putting such meat on shelves suits retailers’ focus on sustainability, lowering CO_2_ emissions, and climate change [31];
A growing interest in market-oriented strategies in the sense that consumers’ reactions are taken into account more and more by food producers. In countries where consumers have a strong interest in high-end or special regional products, the market strategy may gear toward segmenting markets [32];
Annual EU meat consumption is decreasing overall, driven by societal demands, including social, ethical, health, and environmental concerns [33];
A growing demand for pork meat on the international market, especially the Asian market [34], because an outbreak of African swine fever in China wiped out a quarter of the pig population globally, and the price of pork meat is increasing significantly, driving pig producers to pursue economic gains and neglect animal-welfare issues;
A growing market share of meat from entire male pigs;
The public focus on social issues is leaning more toward global warming and general sustainability than a specific animal-welfare issue;
In general, politicians are seeking long-term sustainable solutions rather than short-term solutions, for example, the growth of Green parties in Germany and the Netherlands;

An increase in the sense of a level playing field, referring to a growing wish for harmonization within the EU single market [18].

### 2.4. Step 4 Identify Uncertainties

The fourth step in the scenario construction process is to identify uncertainties that could potentially have an effect on the issue at hand. In the pig castration case, 11 uncertainties were identified. These are now discussed.

The first uncertainty relates to MSs’ actions in banning castration. About one-third of EU countries have banned castration without pain relief [7]. It is uncertain whether the remaining countries will impose similar legislation. Without such legislation, farmers are unlikely to be pressurized into looking for alternatives to castration. In the legislation context, it is also uncertain whether national animal-welfare schemes will incorporate indicators related to pig castration [11].

The second uncertainty relates to consumers’ acceptance of immunocastration. Slaughterhouses and processors particularly expressed a fear that market resistance might deter retailers from accepting meat from immunocastrated pigs [12], mainly because of the shock effects generated by scandals or scares regarding vaccine use in food production [35]. Media, by making false statements about vaccines, may trigger altered purchasing by consumers. Special interest groups may play a role in generating media attention.

The third uncertainty relates to the sharing of costs and benefits. Farmers benefit on average, because the costs of extra vaccine and labor for immunocastrating pigs are outweighed by the benefits linked to higher feed efficiency and thinner carcasses [12]. However, some slaughterhouses would have to bear the extra cost of odor detection for immunocastrated pigs if they believed that the vaccines, if wrongly injected or non-responsive, might be ineffective in eliminating boar taint. Thus, for quality control purposes, slaughterhouses might still have to ensure quality in the slaughter line. The investment in extra quality control might undermine slaughterhouses’ economic benefits [36]. One possibility is that the costs and benefits would be internalized by agreements between slaughterhouses and farmers, or between retailers and slaughterhouses. It is also possible that slaughterhouses might experience cost decreases because of consumers refusing to accept meat from vaccinated pigs [37]. A final possibility is that governments and industry might support farmers with education programs about injecting vaccines correctly [7]. With such training, farmers could handle vaccinations correctly; thus, eliminating the need for slaughterhouses to invest in detection methods.

The fourth uncertainty relates to scientific developments in finding an accurate detection method for tainted carcasses. The search for the automatic detection of boar taint has been in progress for years [38]. If scientists are able to find an automatic and affordable detection method that is 100% guaranteed to detect the two hormones in the slaughter line, this might impact slaughterhouses’ acceptance of entire male pigs as well as immunocastrated pigs [4].

The fifth uncertainty relates to the development of a food tracking system. European countries differ in their investment in tracking and tracing systems that aim to improve information flows for the traceability of food and feed products [39]. From the supply side, efficient information exchange in the system helps agribusiness firms such as slaughterhouses and farmers to reduce disparities and to improve their on-site management [40]. From the consumption side, the application of an advanced food tracking system allows consumers to identify how animals are treated, slaughtered, and prepared, based on which consumers can make informed choices. If EU or MSs’ legislation included the aspect of castration in the tracking system, this might exclude misinformation and enhance consumers’ acceptance of immunocastration. Correspondingly, the supply chain would react to the market acceptance.

The sixth uncertainty relates to how MSs would accept one another’s meat-quality standard. For example, the acceptance level of skatole and androstenone (two compounds related to boar taint) differs between Denmark and Germany [41]. Significant disagreements may disrupt trading relations within the EU. In this respect, MSs’ quality assurance departments play a central role in mutually recognizing one another’s quality scheme (e.g., in 2012, QS in Germany, and IKB in the Netherlands agreed on a common framework for boar taint detection using the Human Nose Scoring (HNS) system [42]).

A seventh uncertainty relates to the control of African swine fever, which is the cause of the current high market demand from Asia, especially China, Vietnam, and the Philippines. The outbreak of swine fever in China has caused a shortage of, and soaring prices for, pork products, forcing China to rely more on imports [34]. In addition, the trade war between China and the US and the political tensions between China and Canada could result in an increasing demand for European/Brazilian pork products [43]. The focus on financial gains distracts producers’ attention from animal-welfare issues. However, the pursuit of economic gains might not be long-lasting because it is not certain whether and when China will be able to control the spread of swine fever, and whether the importation of US/Canadian pork products will be resumed [44]. Both will affect the amount of pork that China will import from Europe. If China reduces its imports, producers may have to look to its internal market, which has a high demand for animal-friendly production systems, for instance banning piglet castration.

The eighth uncertainty, which is related to the seventh, is the acceptance of different castration alternatives in Asian markets. Countries like China have not yet imposed criteria for the castration alternatives. It is uncertain whether new criteria will be added to the trading criteria in the future.

Ninth, there is uncertainty in the European Commission’s political agenda about diverse welfare issues. Several pig welfare issues are lying on the Commission’s table, including tail docking, teeth grinding, and sow stalls [45]. It is uncertain whether the castration issue will be placed under the spotlight. If it is prioritized, legislation may be introduced to specify immunocastration practices and procedures.

Tenth, the general economic climate is included as an uncertainty. It is uncertain how long it will take for Europe and the world to recover from the economic loss consequent to the COVID-19 pandemic and the Russia–Ukraine war. In a negative economic environment, consumers might have less money to spend and reduce their purchasing power for animal-friendly (usually more expensive) products.

Eleventh, there is uncertainty concerning the emphasis on societal concern. Public concern can cover a broad range of social, economic, and environmental issues—including animal welfare [18]. However, concern for a single issue may start to dominate the public debate (e.g., climate change, CO_2_ emissions), thereby reducing attention on a specific animal-welfare issue.

### 2.5. Internal Consistency and Plausibility

The internal consistency and plausibility of the two extreme scenarios were ensured in two ways. First, two authors had several rounds of discussions about the statements and conditions used in the scenario building. Second, a group discussion was held with experts and researchers from the SuSI consortium in December 2019 and January 2021. The statements and conditions were verified and further identified by the expert group. Third, the two scenario scripts were sent to an external expert with more than 35 years of working experience in this field to further evaluate their internal consistency in terms of trends and outcome combinations. Overall, the scenarios were considered internally consistent.

## 3. Results

Two scenarios were sketched out with critical assumptions that uncertainties were scripted into extremely negative and extremely positive storylines. By doing so, we aimed to maximize the variance between scenarios, thereby encompassing the broadest span of possible futures for the implementation of immunocastration in the European market. To create a sense of time in the scenario descriptions, they are narrated in the past tense (looking back on the period 2021–2026) [14]. Relevant stakeholders and identified uncertainties are highlighted in italics.

### 3.1. Scenario 1—All Negative

The SuSI project results were submitted to the EU, and officers in the European Commission read the outcome. Although they were shown that immunocastration is an alternative with both economic and ecological advantages, they decided to leave the market to drive the industry’s transition, because their priority was to revive quickly the European economy after the COVID-19 pandemic and the Russia–Ukraine war and reduce CO_2_ emissions rather than to resolve a particular animal-welfare issue. Thus, there was no pressure on EU MSs to take a more active approach. Even those states or local governments that were positive about the results were not sure how to proceed with legislation and did not want to go it alone.

Asian markets, especially China, were not able to recover their domestic pork supply from the loss caused by African swine fever. Their demand for European pork continued to grow, stimulating European producers to produce as many pigs as possible in a short time. Farmers and processors within the cost-efficient production system formed a strong alliance to lobby their government to focus on the pork sector’s economic benefits rather than on a specific animal-welfare issue. Making adjustments in any type of production system would disrupt their well-established logistics, lower their production efficiency, and consequently harm the sector’s contribution to the national economy in the short term. The piglet castration issue was more and more neglected in that context.

Several public debates in Germany about the piglet issue made Chinese customs officers aware of the market distrust of vaccines. This raised concern among Chinese regulators, who then decided to issue clear instructions about reducing the importation of immunocastrated pigs. Consequently, the international market for immunocastrated pigs became smaller, and farmers and slaughterhouses that produced immunocastrated pigs for Asian markets had to switch to either entire male production or castration with pain relief.

Moreover, the COVID-19 pandemic and the Russia–Ukraine war resulted in countries focusing on resuming major economic activities, bringing a halt to investment in innovating for methods to improve animal welfare, such as innovations for an accurate detection method, in particular for boar taint. Not many breakthroughs for detecting tainted carcasses were achieved. Without absolutely accurate detection methods, big processors and dominant retailers were not sure that the vaccine had been applied effectively. They could not be convinced that there would be no tainted carcasses in the slaughter line. Processors and retailers then tended to lose interest in processing and marketing immunocastrated pigs. Without support from downstream stakeholders, farmers were unlikely to produce immunocastrated pigs. Moreover, a few deaths related to the use of COVID-19 vaccines every now and then raised concern among consumers about the general safety of vaccines. The public became more suspicious about any vaccine’s application in any circumstances. There were more opposing voices about vaccines for immunocastration than before. In addition, Russia’s invasion of Ukraine created global concerns for food shortage, resulting in more producers interested in more economically efficient methods. The example of entire male production in the Netherlands was promoted as a business model. More and more farmers and slaughterhouses saw the economic advantage of entire male production. Even stakeholders in the high-quality-oriented system decided to devise different production techniques to meet the quality requirements with entire males instead of immunocastrated pigs.

In society, most campaigns about societal concerns were targeted at issues relating to health and climate change. Accordingly, leading retailers and food producers were active in participating in, and supporting, environmental movements, such as Greenpeace. Limited attention was paid to animal-welfare issues in general, not to mention the particular issue of piglet castration. Without pressure from society, production chains were not confronted with urgency to change their practice. The number of farmers and slaughterhouses/processors attentive to the issue of piglet castration declined.

In the market, people had less purchasing power because of the loss of income and increasing prices resulting from the economic recession induced by the COVID-19 pandemic and the Russia–Ukraine war. Consumers searched for cheaper products rather than animal-friendly or special regional products. Their interest in ethical products declined in the period between 2021 and 2026. By 2026, throughout Europe, the emphasis was to supply affordable pork products to European consumers. Market segments for animal-friendly products and high quality were rather small. In addition, consumers’ purchasing behavior had changed drastically during the strict lockdown, and online shopping was accepted by many consumers. The trend toward online shopping tended to persist even long after the pandemic. In an online shopping environment, consumers received pushed advertisements or news articles alongside shopping items. At a certain point, influential social media made inaccurate statements about the Improvac vaccine and started a conspiracy theory about the pharmaceutical manufacturer, alleging that harmful chemical residues remained in pork meat. Incorrect information widely shared on social media caused consumers to doubt the food safety of immunocastrated pigs. Producers using vaccines experienced large economic losses. Interested producers were scared away before considering it. By 2026, the market share of immunocastrated pigs became even smaller in the European market; only a few local chains applied the method, as they could communicate directly with consumers about the use of vaccines.

### 3.2. Scenario 2—All Positive

The SuSI project results were submitted to the EU, which later recognized the urgency of resolving the issue because there was a growing tendency for trade disputes between MSs. Resolving the castration issue became a priority task for the EU Commission. Together with the SuSI report, studies about castration reached a large number of audiences including NGOs in different MSs and even those family farms in Eastern Europe. A large number of stakeholders in different production systems started to re-evaluate their business strategies.

In Germany, the legislation banning castration without pain relief was enforced in 2021. Convinced by the SuSI results and pushed by opinion leaders, the German government included the use of vaccines as part of the animal-welfare subsidy scheme. Under the scheme, German farmers could receive financial supports if they took professional training. The scheme signaled a positive cue to farmers, processors, and retailers. Leading retailers indicated clearly that they would accept a certain percentage of pork from immunocastrated pigs. With this declaration by leading retailers, mainstream producers (including farmers, slaughterhouses, and processors) concluded agreements about the specific production procedure for immunocastrated pigs, such as housing conditions, feed composition, times of injections, and so on, and introduced a penalty system linked to boar taint in an effort to lower the risk of ineffective vaccinations.

Germany is one of Europe’s largest pork exporters and importers [46]. Its legislation and its various types of production systems set examples for other countries. Several NGOs in other European countries used the example of German pork industries to lobby the national government, requiring similar actions to improve pig welfare. They launched campaigns in their domestic countries, aiming to raise public awareness of piglet castration as well as to put pressure on the industry and the government [4]. Facing pressure from the EU and the Germans’ interest in immunocastration, MSs’ quality assurance departments were called upon to compile protocols for cross-border trading. In the updated trading schemes, vaccine protocols were included with clear and specific guidelines for making pork products for different types of systems.

Scientists made a breakthrough in finding an automatic method that could accurately identify and sort out tainted meat in the slaughter lines. More and more slaughterhouses and processors implemented the method; thus, they were willing to accept carcasses produced from both entire males and immunocastrated pigs. In combination with the accurate detection method, a penalty system linked to boar taint was introduced by slaughterhouses and processors with cost-efficient and quality-oriented systems; thus, regulating the competition from entire males. In farms with a higher boar-taint rate than other farms, producers realized that the tainted-carcass fine would erode any potential economic benefits. Thus, managers of these cost-oriented farms believed that it was better to turn to the production of immunocastrated pigs.

African swine fever was under control in Asia, and the trading constraint on pork from Canada and America was loosening. The Asian market, especially the Chinese market, had less demand for European pork products. Thus, European producers had to rely more on European consumers, who had higher standards of animal welfare and were more aware of the castration issue. Because immunocastration was proven to be optimal in terms of animal welfare and meat quality, stakeholders in the quality-oriented, animal-welfare-oriented, and regional products systems started to consider immunocastration as an alternative to surgical castration. Some farmers, slaughterhouses, processors, and retailers in these systems cooperated and came up with viable structural plans to further improve piglet welfare and quality, in which immunocastration turned out to be the optimal alternative that could improve animal welfare without lowering meat quality. Additionally, some of them learned from the Netherlands that having NGOs on board would gain more public trust. They therefore worked with local NGOs and put effort into promoting the advantages of immunocastration, thereby gaining consumer confidence and improving their image as socially responsible companies.

The economic climate slowly became positive again, as the EU managed to revive its economy quickly after the pandemic. In a positive economic environment, consumers showed more willingness to pay for products of both higher quality and a higher level of animal welfare, and immunocastration as an alternative that maintained good quality and higher animal welfare turned out to be the most accepted method.

The increasing acceptance of immunocastration resulted partly from the transparent and correct information about immunocastration that consumers received in an online shopping environment. Several leading retailers implemented the transparent tracking system, allowing consumers to read detailed information about the products, including the use of vaccines in pigs and the reasons for using such vaccines. The implementation of a transparent tracking system stopped the spread of false information. By 2026, the production and market acceptance of immunocastrated pigs in Europe became higher than it had been five years previously.

## 4. Discussion

The use of scenario analysis is not without limitations. One major limitation is that the method remains a learning tool; it is not able to predict the future, but it can bind different viewpoints in a systematic manner [18]. Uncertainties and trends are included in scenario building, and managers and policymakers can expand their vision with scenarios, leading to a sharper and more realistic focus on possible futures. Four new insights arose from the scenario analysis.

First and most importantly, the scenario exercise implies that the immunocastration method is unlikely to prevail in the European market. Even when all uncertainties become advantageous for immunocastrated pigs, such as an accurate detection method and a better economic environment, immunocastration faces competition from the method of raising entire males, because the detection method will help to detect tainted carcasses from entire males and divert them to different production lines, and entire males have an economic advantage over immunocastrated pigs [47]. Thus, stakeholders in the cost-oriented production system are unlikely to choose immunocastration over entire males. In this regard, producers who opt for immunocastration might consider integrating into a regional-product chain and emphasize its benefits in terms of higher fat saturation [48]. For this, schemes should be established to build immunocastration into regional products.

Second, actors in our scenarios were not identified by the conventional classification of production stakeholders (e.g., farmers, slaughterhouses, retailers). Instead, drawing on the results from an in-depth multi-country study [4], actors were identified by the orientation of their production system. These were cost-efficiency, quality-oriented, animal-welfare-oriented, specialties/regional products, and import-reliant systems, because stakeholders with the same production function do not always hold the same position toward the castration issue as they are embedded in production systems with different orientations [4] and because the application of alternatives relies not only on adjustments at farm level, such as extra labor and/or vaccine cost, but also on acceptance by slaughterhouses, processors, and retailers along the production chain. As such, this classification has the advantage that the orientation of the system is likely to reveal to motivations of stakeholders; thus, giving the classification relatively more predictive power than the traditional, role-based, stakeholder classifications [4]. A system’s orientation shapes how stakeholders in the system perceive the adjustments necessitated by the castration issue. Stakeholders within a particular production system tend to develop agreements regarding how the issue should be handled along the entire production process. Consequently, grouping chain actors by their embedded production systems is appropriate for scenario analysis. Accordingly, a further development of the immunocastration market will depend on the outcomes of a number of uncertainties and trends imposed on these production systems. The uncertainties include the economic situation in Europe and the world after COVID-19 and the Russia–Ukraine war, the role and positions adopted by NGOs in individual European countries, and whether a sophisticated detection method will materialize. Although developing a more sophisticated detection method would stimulate the production chain to re-evaluate both immunocastration and the entire male method, reassessing and distributing costs and benefits may present a challenge to the industry.

Third, several recent studies from the social perspective have focused on consumers’ sensory preferences [37,38], attitudes, and purchasing behavior regarding pork [13], emphasizing that producers need to satisfy consumers’ needs and wants [11]. However, most consumers are not well informed about the issues [11]. Although the scenarios suggest that consumers are important uncertainty factors, they represent only some of the uncertainties related to the implementation of immunocastration and improvement in pig welfare. They also are influenced by complex patterns of interacting factors in the environment, and their perceptions toward immunocastration and the other alternatives are likely to be influenced by uncertainties around them. Farmers, slaughterhouses, processors, retailers of different production systems, the government, and NGOs are the main drivers of interest in the issue. The characteristics of production systems [4], stakeholders’ different positions, the role of the legislative body, and the certification organization for animal-friendliness or meat quality should also be a focus of future research.

Finally, our scenarios provide valuable implications for policymakers with a concern for animal welfare. To ensure that pigs and pig products can be traded with the fewest barriers possible, the European Commission initially preferred the adoption of a single solution for the entire EU [4]. However, the scenarios presented here suggest that arguing for “the one and best solution” is unlikely to have a promising end, because different systems have been co-existing, and will continue to co-exist, regardless of local/regional/international markets. Different systems will thus have to find different solutions in different development scenarios. Therefore, the debate among stakeholder groups should focus on how to coordinate efficiently between different production systems to supply products that can satisfy different market demands in terms of cost, high quality, regional production, and animal friendliness. For this issue in particular, policymakers should pay more attention to production systems as actors rather than to stakeholder groups. Improvements in pig welfare in terms of ending castration are likely to result from well-matched supply and demand. That is, aligning production systems with consumers’ demands in terms of orientation (e.g., region, quality, price, or ethics) seems to be a more realistic path. Researchers may thus help to identify market segments according to consumers’ motives. Therefore, establishing an organization to coordinate the interests of different stakeholders and different production systems may help to create a more positive outcome for the acceptance of immunocastration and the general improvement in pig welfare.

## 5. Conclusions

By conducting a scenario analysis, our study provided insights into variances in possible futures for implementing immunocastration. Immunocastration is unlikely to become a single solution to replace surgical castration of piglets. European pork production systems vary in their orientation regarding animal welfare, quality, cost, type of products, and trading relations, and all these factors complicate the supply and demand for pork meat. Therefore, instead of debating which method is the one and the best for the EU single market, our study calls for the co-existence of all alternatives and the development of protocols for these existing alternatives to fit pork production systems with different orientations.

## Figures and Tables

**Table 1:** Actors, pre-determined elements, and uncertainties included in the scenario analysis regarding future pig castration.

Actors	Pre-Determined Elements	Uncertainties
Cost-efficiency systems (e.g., NL, DK, SP, Cooperl in FR, northern DE)	Economic advantage	The sharing of costs and benefits
Quality-oriented systems (e.g., FR, IT)	Quality advantage	Consumer acceptance
Animal-welfare-oriented systems (e.g., SE, NO)	Animal-welfare advantage	Political agenda
Specialties/regional products systems (e.g., southern DE, SI, FR local chains)	Quality advantage Special products	Quality standard
Import-reliant systems (e.g., SL, BG)	Market competitiveness Product differentiation Price advantage	Economic climate Acceptance by major markets
Governments	Animal-welfare policies Level playing field	Political agreement Country’s actions in banning castration
Scientists	Scientific validation of alternatives	Search for an accurate detection method
Special interest groups	Attention on the castration issue	Emphasis on societal concern
Veterinarians	Scientific validation of alternatives	Scientific validation
Media	Attention on the castration issue	Scandals and scares

## Appendix A

**Table A1:** Steps in scenario construction.

1	Define the issues you wish to understand better in terms of time frame, scope and decision variables. Review the past to get a feel for degrees of uncertainty and volatility.
2	Identify the major stakeholders or actors who would have an interest in these issues, both those who may be affected by it and those who could influence matters appreciably. Identify their current roles, interests and power positions.
3	Make a list of current trends or predetermined elements that will affect the variable(s) of interest. Briefly explain each, including how and why it exerts an influence. Constructing a diagram may be helpful to show interlinkages and causal relationships.
4	Identify key uncertainties whose resolution will significantly affect the variables of interest to you. Briefly explain how these uncertain events matter, as well as how they interrelate.
5	Construct two forced scenarios by placing all positive outcomes of key uncertainties in one scenario and all negative outcomes in the other. Add selected trends and predetermined elements to these extreme scenarios.
6	Next assess the internal consistency and plausibility of these artificial scenarios. Identify where and why these forced scenarios may be internally inconsistent (in terms of trends and outcome combinations).

Source: Adapted from Schoemaker [14] and Ingenbleek et al. [18].
